# Investigation of Parameters Affecting the Equivalent Yield Curvature of Reinforced Concrete Columns

**DOI:** 10.3390/ma13071594

**Published:** 2020-03-31

**Authors:** Umut Hasgul

**Affiliations:** Department of Civil Engineering, Balikesir University, 10145 Balikesir, Turkey; hasgul@balikesir.edu.tr; Tel.: +90-266-6121194 (ext. 123202)

**Keywords:** equivalent yield curvature, reinforced concrete column, moment–curvature analysis, axial load level, reinforcement ratio, concrete compression strength

## Abstract

In this study, the response quantities affecting the equivalent yield curvature, which is important in the deformation-based seismic design and assessment of structural systems, are investigated for reinforced concrete columns with a square cross-section. In this context, the equivalent yield curvatures were determined by conducting moment–curvature analyses on various column models, in which the axial load level, cross-section dimension, longitudinal reinforcement ratio, and concrete compression strength were changed parametrically, and the independent and/or combined effects of the relevant parameters were discussed. Depending on the axial load levels of *P*/*A_g_f_c_*′ < 0.3, *P*/*A_g_f_c_*′ = 0.3, and *P*/*A_g_f_c_*′ > 0.3 for the considered columns, the yielding of reinforcement, yielding of reinforcement and/or concrete crushing, and concrete crushing governed the yield conditions, respectively. It can be noted that the cross-section dimension and axial load level became the primary parameters. Even though the independent effects with regard to particular parameters remained at minimal levels, the combined effects of them with the axial load became important in terms of the equivalent yield curvature.

## 1. Introduction

One of the most important problems in force-based design is the selection of an appropriate stiffness, especially for reinforced concrete building and bridge-type construction systems. The stiffnesses used when determining the natural vibration period of structures and linked to the equivalent lateral earthquake loads are not known at the beginning of design. The gross-section stiffness and reduced effective stiffness considering the cracking of concrete in some situations are based on structural members (such as beams, columns, shear walls, etc.). Many design codes stipulate coefficients for reducing the bending stiffness of different types of members [1,2,3,4,5]. Even though this approach represents a significant improvement in the use of gross-section properties, an adequate approximation in determining the dynamic behavior of systems cannot be provided since the possible influences, such as the axial load ratio, longitudinal reinforcement ratio, and material characteristics, on the stiffness are not considered [6,7]. Within the force-based design concept, while the member’s stiffness directly affects the period of the structure, lateral load distribution, and displacement demand, it becomes more of an issue in determining the yield displacement and hence displacement ductility within the framework of nonlinear static or dynamic analyses [8]. It should be noted that despite the effective stiffness expressions recommended in the current design codes, these may lead to inadequate predictions of deformation demands within the performance-based design and assessment concept [9].

**Figure 1:**
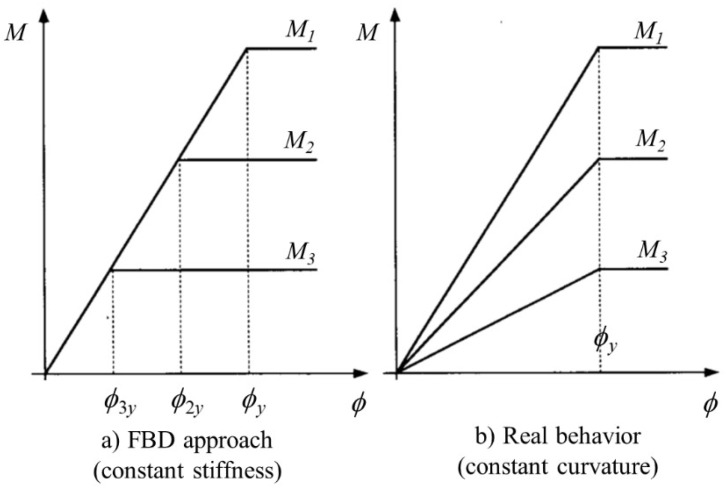
Influence of strength on the moment–curvature response (**a**) constant stiffness, and (**b**) constant curvature approaches [6,7].

This approach, with regard to the bending stiffness, points out not only that the effective stiffness is constant, regardless of the strength, but also that the stiffness does not change with the increasing bending capacity of the member. Therefore, the yield displacement becomes directly proportional to the strength, as shown in Figure 1a. Research conducted by others [6,7,10] has demonstrated that the bending stiffness is directly proportional to the member strength and hence the constant stiffness approach becomes invalid. As shown in Figure 1b, this outcome revealed the use of a constant yield curvature. Parametric investigations within the limits stipulated in the design specifications demonstrated that the yield curvature is largely dependent on the cross-sectional characteristics, but independent of the strength (Figure 1b). As a consequence, the equivalent yield curvature (Δ*_y_*) obtained from bi-linearization of the moment–curvature response is almost constant in very large regions of the axial load level and longitudinal reinforcement ratio [6,7,10].

**Figure 2:**
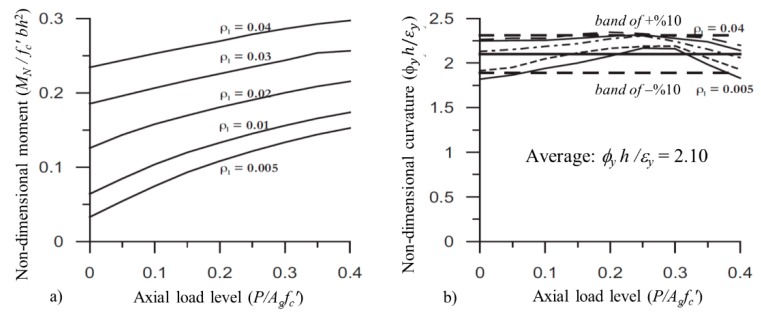
In non-dimensional form (**a**) moment capacities, and (**b**) equivalent yield curvatures [6].

Priestley [6] investigated several parameters affecting the effective bending stiffness and yield curvature of rectangular cross-sectional columns. In the study, the various moment–curvature analyses were conducted for particular axial load levels (*P*/*A_g_f_c_*′ = 0–0.4) and reinforcement ratios (*ρ_l_* = 0.5%–4.0%) under a constant dimension, thickness of concrete cover, concrete compressive strength, and yield strength of reinforcement. The results showed that both parameters had a significant influence on the moment carrying capacity, as would be expected (Figure 2a). However, the dimensionless yield curvatures (*ϕ_y_h*/*ε_y_*) were slightly affected by the changes in relation to these parameters, and the mean value of 2.10 was recommended for the rectangular column sections. Therefore, the equivalent yield curvatures may be determined within a band of ±10% (Figure 2b).

Li [11] studied the non-linear behavior of reinforced concrete columns subjected to cyclic lateral loads. After conducting various moment–curvature analyses, they deduced that the axial load level of members significantly changed the yield curvature. Here, the yield condition occurred with the yielding of outermost tension reinforcement to the neutral axis for *P*/*A_g_f_c_*′ ≤ 0.2, and the equivalent curvature increased as the axial load increased. For the further axial load levels, the curvature showed a decreasing trend, since the concrete crushing in the extreme compression fiber governed the yield condition.

Sheikh et al. [12] evaluated the equivalent yield curvatures of a large number of normal- and high-strength reinforced concrete columns by referencing the moment–curvature analysis results. They reported that the yield curvature was not significantly influenced by the section size (diameter), axial load level, amount of longitudinal reinforcement, concrete compressive strength, and thickness of concrete cover. However, the importance of the reinforcement ratio was emphasized in the scope of the accurate performance evaluation of columns. Montes and Aschleim conducted a series of moment–curvature analyses on both circular and rectangular column cross-sections to realistically determine the yield displacement [13]. In the study, the axial load level, cross-section height, reinforcement ratio, yield strength of reinforcement, and concrete compressive strength were parametrically changed, and they indicated that despite the fact that the concrete compressive strength did not provide an apparent impact, the parameters of the reinforcement ratio and yield strength had significant impacts on the yield curvature. Zhao et al. [14] evaluated the response parameters influencing the yield curvature of high-strength concrete shear walls. The moment–curvature analyses showed that the factors most influencing the yield curvature are the yield strain of reinforcement and level of axial load ratio. Avsar et al. [15] also pointed out similar parameters. Tjhin et al. [16] studied the equivalent yield curvature for the ductile design of reinforced concrete shear wall buildings. Various numerical analyses, including those on the axial load level, reinforcement ratio, concrete compressive strength, and yield strength of reinforcement, showed that the contributions of the concrete compressive strength and web reinforcement ratio to the yield curvature remained at minimal levels. Wang et al. [17] conducted a comprehensive parametric study on almost 5000 T-shaped wall cross-sections based on the regression analyses of numerical results. In the case of the tensile reinforcements determining the yielding condition at lower axial load levels, the yield curvature increases with the increase of the axial load ratio. On the contrary, when compressive concrete governs the yielding condition at higher axial load levels, the yield curvature decreases. Their study also indicated that the while yield curvature had an increasing trend with the amount of longitudinal reinforcement, the yield curvature decreased with the concrete strength, regardless of the yielding condition. Recently, Hasgul [18] numerically investigated the usability of practical equations proposed in Priestley [6,7] and Montes and Aschleim [13] for various column models, in which the axial load level, cross-section dimension, reinforcement ratio, and concrete compression strength were diversified parametrically. Considering the variations in each independent parameter, the practical equations have good correlations with the moment–curvature analysis results as long as the yielding of longitudinal reinforcement governs the yielding condition. However, they may be inadequate in determining the combined effects of them with an increasing axial load level (*P*/*A_g_f_c_*′ > 0.3).

The results of numerical investigations related to reinforced concrete columns in the literature [6,14,17,19,20,21,22] show that the cross-section dimensions, axial load, reinforcement ratio, and characteristic strengths of concrete and steel reinforcement have significant influences on the equivalent yield curvature, and thus on the yield displacement and displacement ductility. It was thought that an investigation of the independent and/or combined effects of these parameters would be important for achieving accurate predictions of plastic deformation demands within the framework of nonlinear static or dynamic analyses.

In the present study, different response quantities affecting the equivalent yield curvature were numerically investigated for square reinforced concrete columns. In this context, over 1500 column models were generated by parametrically changing the dimensions, axial load level, longitudinal reinforcement ratio, and concrete compressive strength. The changes with respect to the yield strength of reinforcement were not taken into consideration since the yield curvature was directly influenced by it. The independent and/or combined effects of these parameters on the equivalent yield curvature were discussed after conducting moment–curvature analyses.

## 2. Numerical Investigation on the Reinforced Columns

### 2.1. Properties of Column Cross-Sections

**Figure 3:**
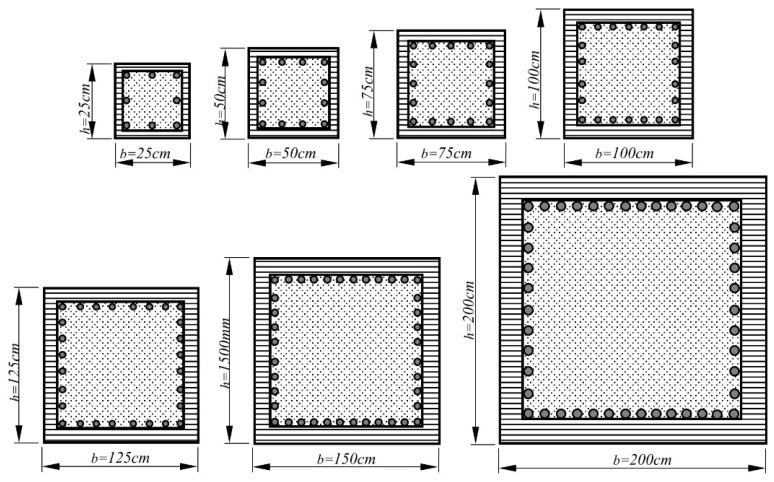
Reinforced concrete column cross-sections.

In the scope of numerical analyses, the upper and lower bands with regards to different response parameters of reinforced concrete column sections were selected in order to represent common practical design applications. Therefore, all possible combinations corresponding to the analysis parameters, which play a major role in determining the curvature response of column sections, have been considered to discuss how and in what proportion these parameters change the curvatures. A total of 56 reinforced concrete column models, of which seven square cross-sectional dimensions (*bxh*) and eight longitudinal reinforcement ratios (*ρ_l_*) were systematically changed, were generated to investigate the response quantities that have an influence on the equivalent yield curvature. Thereafter, these column models were diversified by changing the axial load levels (in the *P*/*A_g_f_c_*′ range of 0 to 0.5) and concrete compressive strengths (ranged from 20 to 60 MPa). In this manner, a total of 1680 analysis models were developed. The geometries of analyzed column sections are given in Figure 3, and the range of variation for each response parameter is summarized in Table 1. Since the yield curvatures concerning the column sections are directly affected by the yield strength of reinforcement (*f_y_*) and the cover thickness ratio has a notable influence, these parameters were kept constant to sensitively consider the independent and/or combined effects of variations in other parameters (Table 1).

### 2.2. Determination of Equivalent Yield Curvature

Moment–curvature analysis is required to determine the behavior of a reinforced concrete cross-section in the cracking, elastic, and plastic regions; to determine the deformation demands in specific limit states (such as serviceability, damage control, and life-safety) between the yield and ultimate conditions; and to determine the force-deformation-ductility relationships of members. However, moment–curvature analysis is also a useful tool for checking whether or not tension reinforcements are ruptured, compression reinforcements are bucked, compression concrete block is crushed, and the member’s shear capacity is exceeded for the stipulated limit state [23].

**Figure 4:**
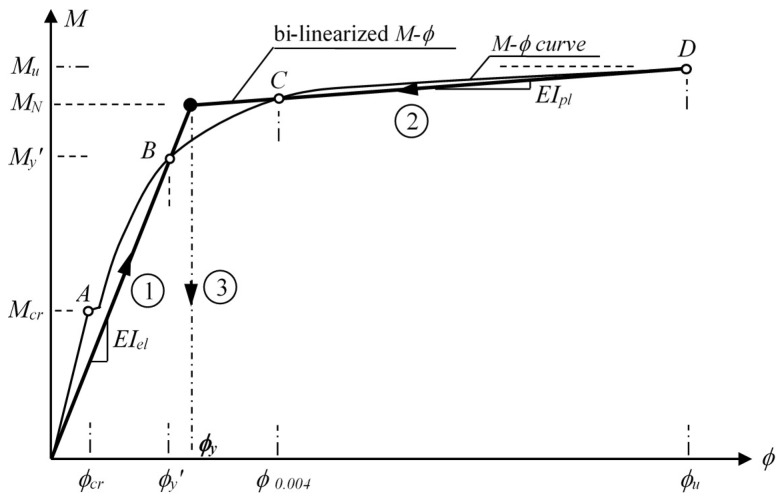
Bi-linearization of a typical moment–curvature response.

For the ductility calculation at a cross-section level, the moment–curvature response can be idealized by two linear branches comprised of the elastic and plastic sections. As shown in Figure 4, the first linear branch of the bi-linear response occurs from the origin to the first yield point B (*ϕ_y_*′, *M_y_*′). Later on, the equivalent yield curvature (*ϕ_y_*) related to the idealized response can be determined by extrapolating the line up to the nominal moment capacity (point C). Therefore, the plastic branch of the response comes into view when combining the equivalent yield point (*ϕ_y_*, *M_N_*) and ultimate point (*ϕ_u_*, *M_u_*) (Figure 4). The equivalent yield curvature, and elastic and plastic stiffnesses (*EI_el_* and *EI_pl_*, respectively) with respect to the bi-linearized moment–curvature response can be calculated by means of Equation (1)–(3), respectively [7].

(1) $$\phi_{y}=\frac{M_{N}}{M_{y}{}^{\prime }}\phi_{y}{}^{\prime }$$

(2) $$EI_{el}=\frac{M_{y}{}^{\prime }}{\phi_{y}{}^{\prime }}=\frac{M_{N}}{\phi_{y}}$$

(3) $$EI_{pl}=\frac{M_{u}-M_{N}}{\phi_{u}-\phi_{y}}$$

### 2.3. Investigation of Response Quantities Affecting the Equivalent Yield Curvature

In this section of the study, the particular response quantities (axial load level—*P*/*A_g_f_c_*′, cross-section dimension—*bxh*, longitudinal reinforcement ratio—*ρ*_l_, and concrete compressive strength—*f_c_*′), which may directly or indirectly affect the equivalent yield curvature, were numerically investigated for various column sections. In this context, moment–curvature analyses were conducted on the constituted column models, and the equivalent yield curvatures were calculated based on the approach indicated above. The variations of yield curvatures from the point of the considered parameters, as well as how and in what proportion these parameters change the curvatures, were discussed comprehensively. The moment–curvature analyses were conducted by means of the *CUMBIA* computer program [24]. *CUMBIA*, which was coded through the *Matlab* platform, can perform nonlinear cross-section analyses of rectangular and circular reinforced concrete column sections separately considering the cover and core layers. In this sense, constitutive models for un-confined and confined concrete, as well as reinforced steel bars, can be specified by the user or default models in *CUMBIA*. While the default models used for the un-confined and confined concrete are those of Mander et al. [25,26], two models for the reinforced steel bars can be applied: that of King et al. [27] and that of Raynor et al. [28]. In addition, the force-deformation-displacement-ductility relationships corresponding to any limit state (such as serviceability, damage control, life-safety, and so on) can be determined for an axial load and single or double bending. The shear deformations and onset of buckling can also be computed by the procedures described in the references [10,29] and [30,31], respectively. In the moment–curvature analyses of this study, the un-confined and confined concrete models recommended by Mander et al. [25,26] were used for the cover and core layers. King and others’ stress–strain model [27] was applied for the deformed reinforcement steel bars.

In the determination of the yield condition relation to the investigated column cross-sections, the axial load levels governed the behavior, irrespective of whether the yield of reinforcement yielded (*ε_s_* = *ε_y_*) and/or the outermost cover concrete layer crushed (*ε_c_* = *ε_co_*). Therefore, the following cases, in general, came into existence in the formation of yield curvatures, depending on the axial load level:

*P*/*A_g_f_c_*′ < 0.3 for the yield of steel reinforcement;

*P*/*A_g_f_c_*′ = 0.3 for the yield of steel reinforcement and/or concrete crushing;

*P*/*A_g_f_c_*′ > 0.3 for the concrete crushing.

Accordingly, discussions with respect to the considered response parameters were conducted for two particular zones: *P*/*A_g_f_c_*′ ≤ 0.3 and *P*/*A_g_f_c_*′ > 0.3. The variations of yield curvatures with the related parameters and the comparisons were presented for the constant yield strength of reinforcement. It is well-known not only that the general characteristics belonging to the response will not change in the cases of lower or higher yield strength reinforcements, but also that the equivalent curvature will increase as the yield strength increases [6,7,10,13]. The influence of each parameter on the curvature response was evaluated by using the median values statistically.

#### 2.3.1. Effect of the Axial Load Level

**Figure 5:**
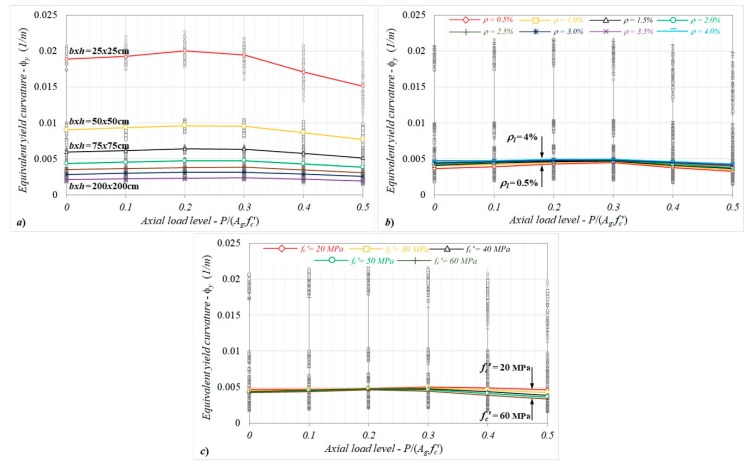
The variations of equivalent yield curvatures with the axial load level in terms of different (**a**) column dimensions, (**b**) reinforcement ratios, and (**c**) concrete compressive strengths.

After conducting moment–curvature analyses for the column cross-sections, the variations of equivalent yield curvatures with the axial load levels (*P*/*A_g_f_c_*′) were investigated, and are given in Figure 5a–c from the points of view of different section dimensions, longitudinal reinforcement ratios, and concrete compressive strengths. 

When the influence of the axial load level on the equivalent yield curvature was investigated (Figure 5a) in terms of different column dimensions, the yield curvature somewhat increased (<10%) to *P*/*A_g_f_c_*′ ≤ 0.3. For the further axial load levels (*P*/*A_g_f_c_*′ > 0.3), in which the concrete crushing governed the yield condition, conversely, the curvatures decreased up to ≈20% in comparison to the case without an axial load. It can be noted that despite the relative changes in the yield curvature for *P*/*A_g_f_c_*′ ≤ 0.3 having similar ratios for each column dimension, the differences showed a decreasing trend as the cross-section dimension increased, as shown in Figure 5a.

For different reinforcement ratios ranging from 0.5% to 4.0%, as shown in Figure 5b, the equivalent curvature displayed an increasing tendency as the axial load level increased up to 0.3, and this inclination turned into a decreasing trend for the further *P*/*A_g_f_c_*′ levels. It can be noted that an increase in the axial load level results in a more pronounced influence for low reinforcement ratios, and the curvature can decrease by up to 22% in comparison to the case without an axial load.

When the analysis results were taken into consideration in terms of the concrete compressive strength, as shown in Figure 5c, despite the equivalent yield curvatures increasing by up to 10% for *P*/*A_g_f_c_*′ ≤ 0.3, they exhibited a decreasing trend for axial load levels preceding the concrete crushing, depending on the compressive strength. In this region, while the relative changes were limited by −1.2% in comparison to the non-axial load cases, the differences calculated could reach up to −22% for the case of *f_c_*′ = 60 MPa. This outcome means that the combined effect of the axial load and concrete compressive strength on the equivalent yield curvature is important.

#### 2.3.2. Effect of the Cross-Sectional Dimensions

**Figure 6:**
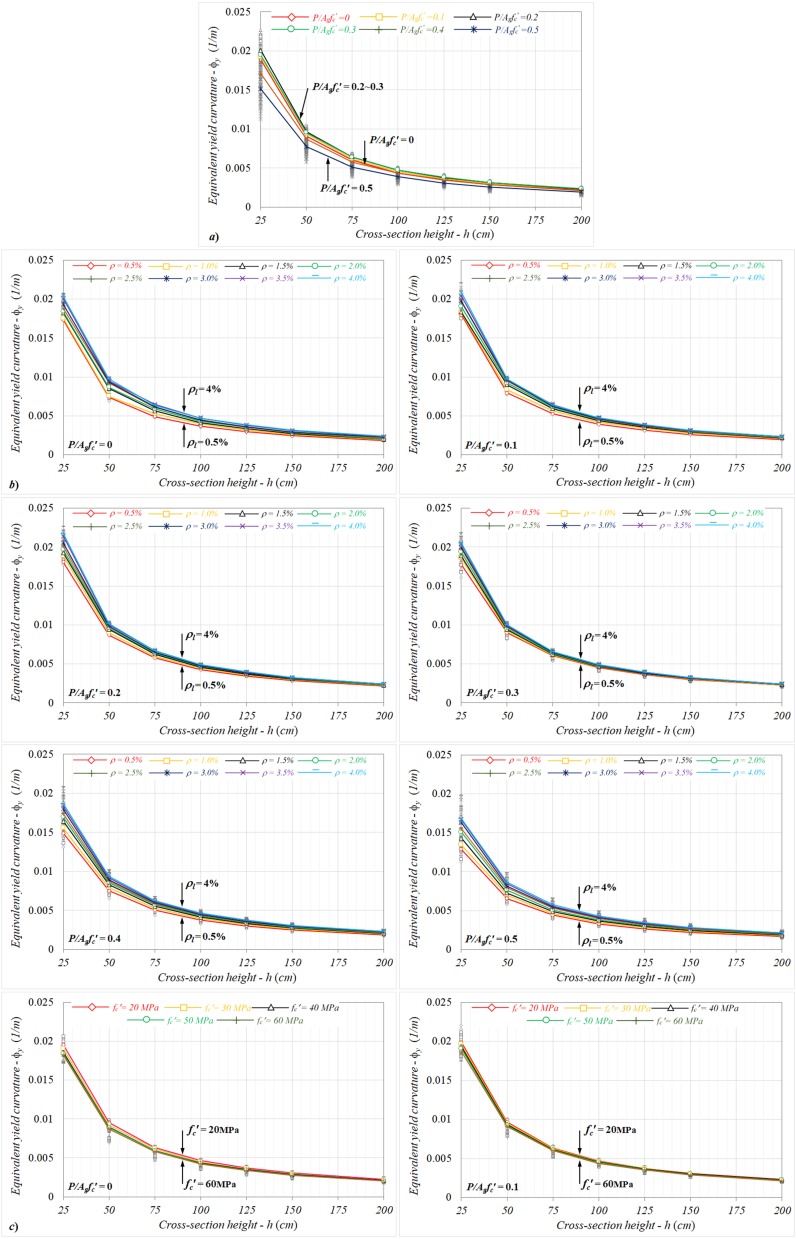

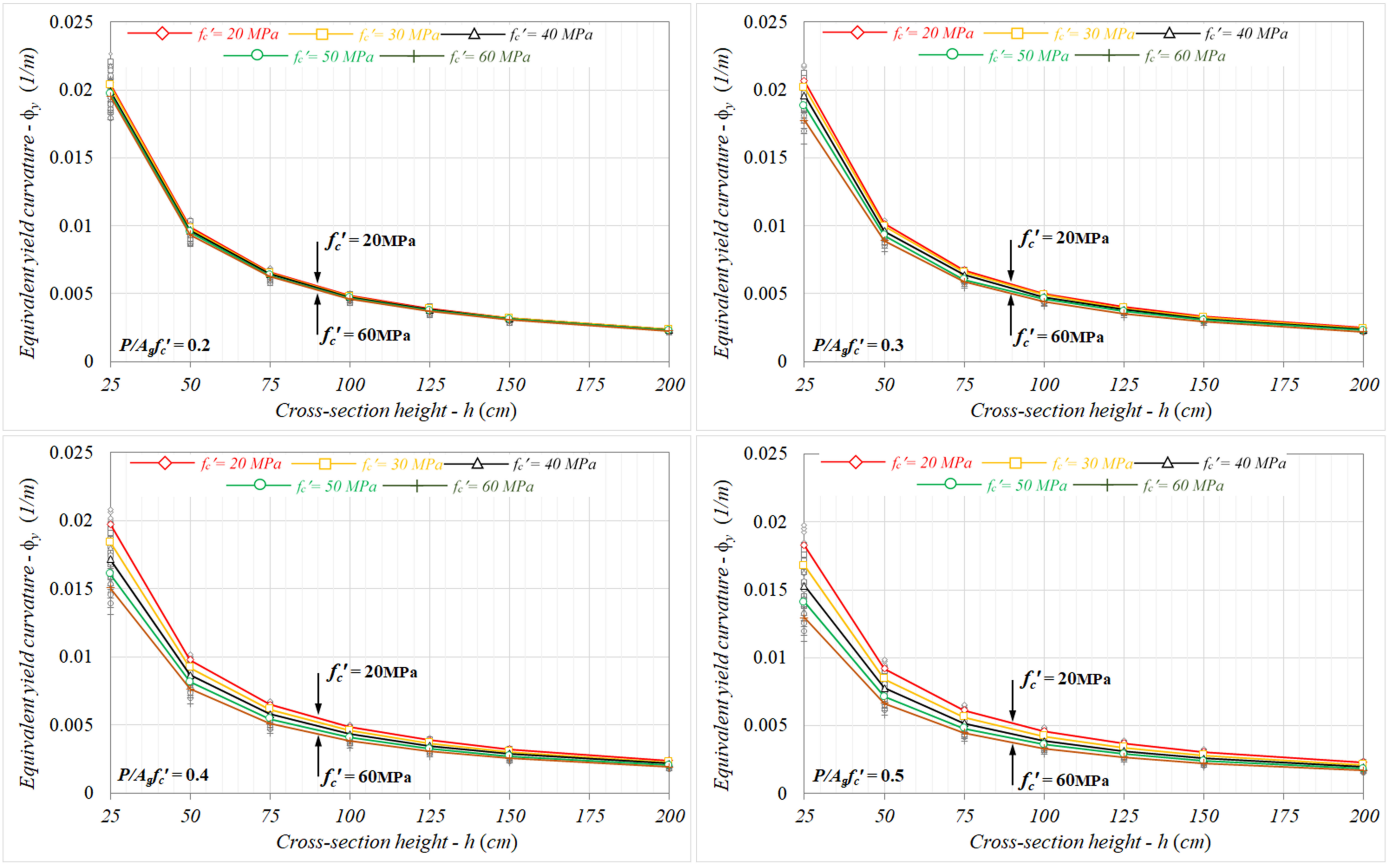
The variations of equivalent yield curvatures with the cross-section height in terms of different (**a**) axial load levels, (**b**) reinforcement ratios, and (**c**) concrete compressive strengths.

For the square column sections ranging from *bxh* = 25 × 25 to 200 × 200 cm, the variations of the equivalent yield curvature with the cross-sectional height (*h*) are given in Figure 6a–c from the perspective of the particular axial load levels, longitudinal reinforcement ratios, and concrete compressive strengths. 

Considering the variations of equivalent yield curvatures determined for the particular cross-sectional heights in each axial load level, although the equivalent curvatures rapidly decreased up to the section height of 125 cm, the changes remained limited for further dimensions (Figure 6a). Here, while the equivalent curvatures between two lower section heights (*h* = 25–50 cm) relatively decreased by nearly 51%, these changes among *h* = 50–75, 75–100, 100–125, and 125–150 cm were roughly determined to be 33%, 26%, 20%, and 18%, respectively. It can be noted that the relative changes calculated for the equivalent yield curvature had similar ratios for all axial load levels.

Regardless of the yield of reinforcement and/or concrete crushing regarding the yield condition, an increase or decrease in the reinforcement ratio for different sectional heights did not affect the characteristics of curvature behavior, as shown in Figure 6b. It was also noted that the relative changes between the equivalent curvatures calculated for the increasing section heights were nearly the same for each reinforcement ratio. Based on the analysis results, the combined influences of the column dimension and longitudinal reinforcement ratio had no significant impact on the equivalent yield curvature. 

Similar to the response obtained from the reinforcement ratio, the direct effect of the concrete compressive strength on the yield curvature response was determined to be very small, as shown in Figure 6c. Depending on the increasing section height, it can be seen that the relative change ratios calculated between the yield curvatures were pretty close to each other for the particular concrete compressive strengths between *f_c_*′ = 20 and 60 MPa.

#### 2.3.3. Effect of the Longitudinal Reinforcement Ratio

**Figure 7:**
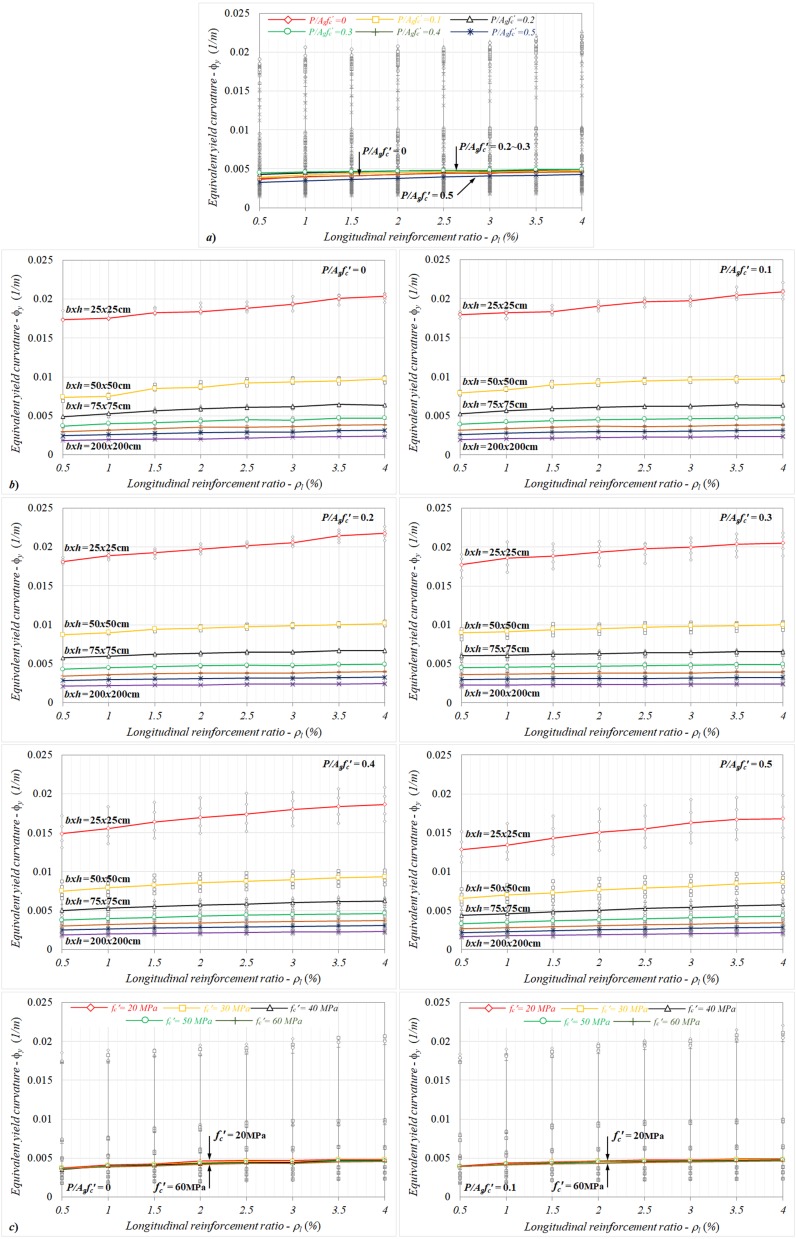

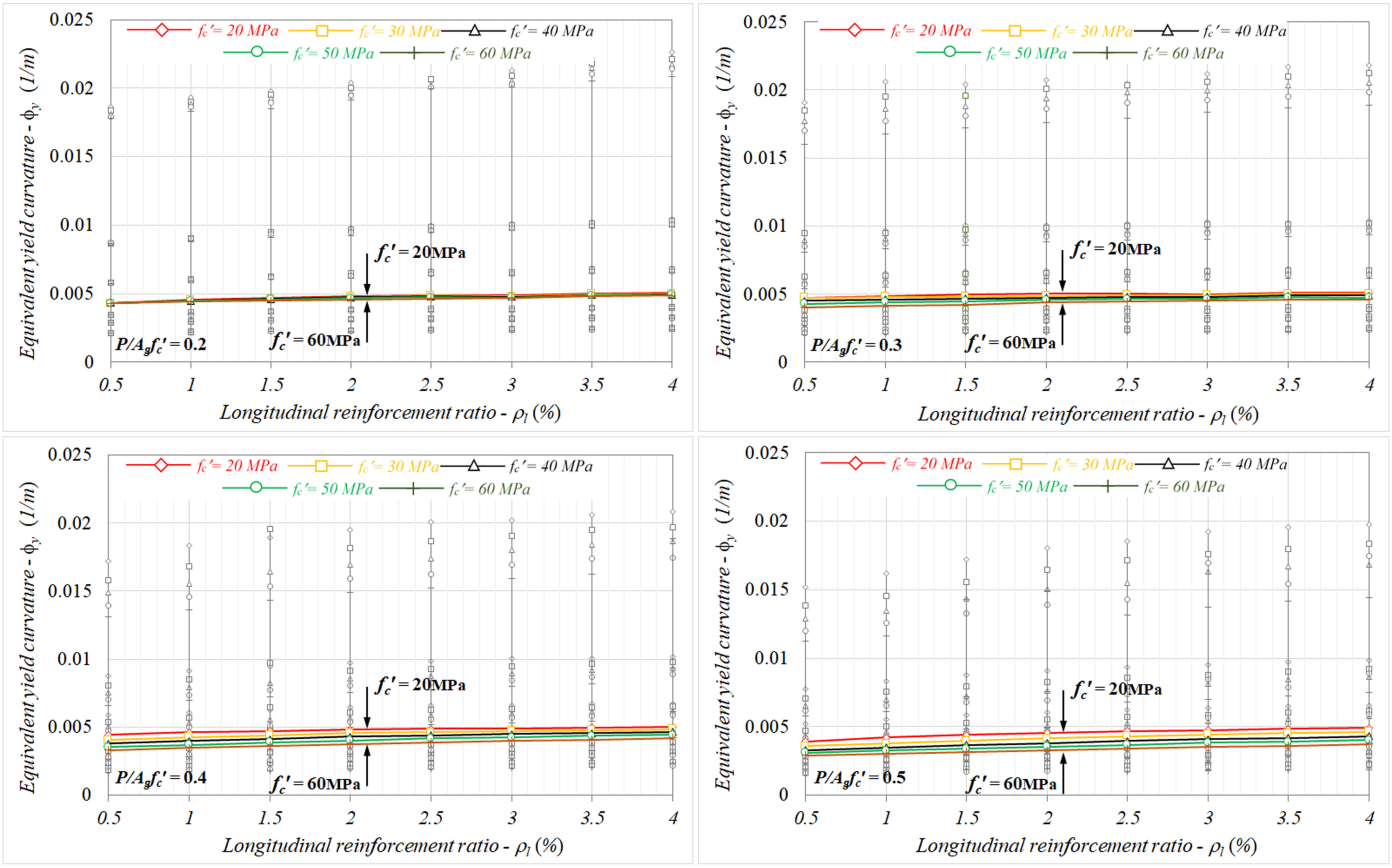
The variations of equivalent yield curvatures with the reinforcement ratio in terms of different (**a**) axial load levels, (**b**) column dimensions, and (**c**) concrete compressive strengths.

Based on the particular longitudinal reinforcement ratios between the range of *ρ_l_* = 0.5% and 4%, the variations of equivalent yield curvatures with the reinforcement ratios were investigate and are given in Figure 7a–c from the standpoint of the axial load levels, column dimensions, and concrete compressive strengths. For each considered axial load ratio, the equivalent yield curvature showed an increasing trend as the reinforcement ratio increased in the column section, as shown in Figure 7a. By referencing the curvature value calculated for the smallest reinforcement ratio of *ρ_l_* = 0.5%, relative changes up to 31% were determined, depending on the axial load level.

It can be noted that the general characteristic corresponding to the equivalent yield curvature–reinforcement ratio responses did not change for different column sections (Figure 7b). However, the calculated relative changes with increasing reinforcement ratios had similar ratios for all column sections. It should be noted that despite the combined effect of the reinforcement ratio and section dimension on the equivalent yield curvature not being notable, as shown in Figure 7b, the relative changes calculated for each *P*/*A_g_f_c_*′ level showed significant differences. Here, the equivalent curvatures corresponding to *P*/*A_g_f_c_*′ = 0, 0.1, 0.2, and 0.3 could relatively increase by up to 28%, 21%, 16%, and 10%, respectively, depending on the reinforcement ratio. For the further axial load levels, the related differences reached up to 31% in comparison to the results calculated for *ρ_l_* = 0.5%.

For lower axial load ratios (*P*/*A_g_f_c_*′ ≤ 0.3), where the reinforcement yield governed the yield condition, as shown in Figure 7c, the reinforcement ratio–curvature responses obtained for different concrete compressive strengths were pretty close to each other (less than 10%). In contrast to this behavior, in the case of further axial load levels (*P*/*A_g_f_c_*′ > 0.3), the yield curvatures decreased as the concrete compressive strength increased for each reinforcement ratio. It can be noted here that the general characteristic relation to the curvature responses did not have a significant influence. However, the equivalent curvatures exhibited an increasing trend as the reinforcement ratio increased for each compressive strength (Figure 7c). It can also be noted that the relative changes may increase up to 30% between the curvatures calculated for the smallest and largest reinforcement ratios (*ρ_l_* = 0.5% and 4%), depending on the concrete compressive strength. Here, the combined effect of the axial load ratio and compressive strength became evident for the each reinforcement ratio.

#### 2.3.4. Effect of the Concrete Compressive Strength

**Figure 8:**
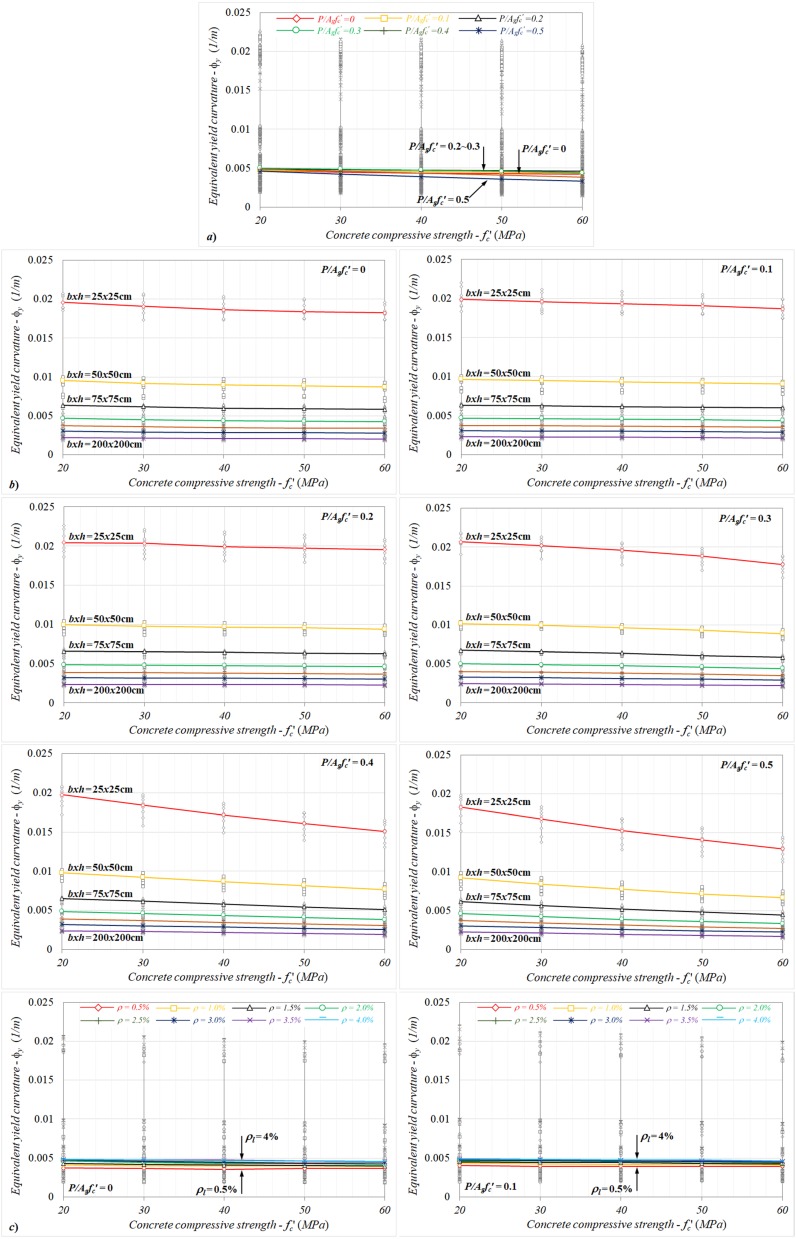

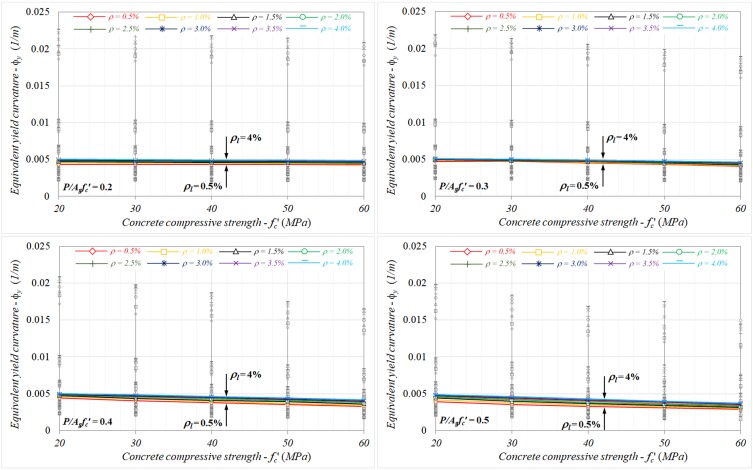
The variations of equivalent yield curvatures with the concrete compressive strength in terms of different (**a**) axial load levels, (**b**) column dimensions, and (**c**) reinforcement ratios.

For the column sections with different concrete compressive strengths ranging from *f_c_*′ = 20 to 60 MPa, the variations of equivalent yield curvatures with the compressive strengths were investigated and are given in Figure 8a–c from the perspective of different axial load levels, column dimensions, and reinforcement ratios. While the direct effect of the concrete compressive strength on the yield curvature response was determined to be very small (less than 10%) up to *P*/*A_g_f_c_*′ < 0.3, as shown in Figure 8a, the curvatures rapidly decreased as the compressive strength increased for the further axial load levels (*P*/*A_g_f_c_*′ ≥ 0.3), where the concrete crushing governed the yield condition. In this region, the differences between the yield curvatures calculated for *f_c_*′ = 20 and 60 MPa may reach up to 28%. 

Referring to Figure 8b, the yield curvatures rapidly decreased, as would be expected, as the section height increased, regardless of other quantities. The relative changes in the yield curvature remained limited (less than 10%) as the concrete strength increased for *P*/*A_g_f_c_*′ < 0.3. In contrast to this behavior, the combined effect of the axial load and concrete strength on the equivalent yield curvature was important for the further axial load levels. In this sense, the differences between the yield curvatures for the column models with *f_c_*′ = 20 and 60 MPa were roughly determined to be max. −14%, −24%, and −29% at *P*/*A_g_f_c_*′ = 0.3, 0.4, and 0.5, respectively. It can also be noted that these differences were nearly the same for each column dimension. When the graphs related to the concrete compressive strength–yield curvature responses were investigated for different reinforcement ratios (Figure 8c), it was seen that the equivalent curvatures increased by an amount as the reinforcement ratio increased. Similar to the response obtained for the column dimension, for *P*/*A_g_f_c_*′ < 0.3, the changes of yield curvatures were less than 10%, even when the concrete strength increased in all reinforcement ratios, indicating that there is no notable interaction between the concrete compressive strength and reinforcement ratio in the equivalent yield curvature. Conversely, the yield curvatures showed a rapidly decreasing character for *P*/*A_g_f_c_*′ ≥ 0.3, as the compressive strength increased, as shown in Figure 8c. As a consequence of the yield curvatures calculated between *f_c_*′ = 20 and 60 MPa, the relative changes calculated at *P*/*A_g_f_c_*′ = 0.3, 0.4, and 0.5 could reach up to max. −15%, −25%, and −28%, respectively. It can be noted that these differences in terms of the compressive strength were roughly the same for each reinforcement ratio.

## 3. Conclusions

**Figure 9:**
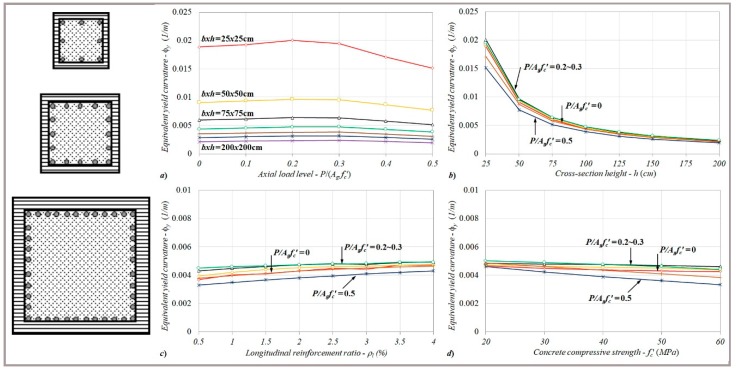
Graphical summary of parameters influencing the equivalent yield curvature.

In this study, the independent and/or combined effects of different response quantities affecting the equivalent yield curvature were numerically investigated for reinforced concrete columns with a square cross-section. A total of 56 reinforced concrete column models, of which seven cross-sectional dimensions and eight longitudinal reinforcement ratios were systematically changed, were generated to discuss how and in what proportion these parameters change the yield curvature. Thereafter, these column models were parametrically diversified by changing the axial load levels (in the *P*/*A_g_f_c_*′ range of 0 to 0.5) and concrete compressive strengths (ranged from 20 to 60 MPa). In this manner, a total of 1680 moment–curvature analysis models were developed with all possible combinations. All results with respect to the parameters influencing the equivalent yield curvature are summarized in a single graph in Figure 9.

When the influence of the axial load level on the equivalent yield curvature was investigated, the yield curvatures slightly increased (less than 10%) up to *P*/*A_g_f_c_*′ ≤ 0.3. For the further axial load levels, the curvatures decreased up to ≈20% in comparison to the non-axial load case. This outcome can be extensively supported by the results of research conducted by others [14,17,20,22,32,33]. However, some researchers [6,12,33,34] have reported that a moderate level of axial compression loads does not significantly affect the equivalent yield curvature. Considering the cross-sectional height—the equivalent yield curvature responses in each axial load level—although the equivalent curvatures rapidly decreased up to the height of 125 cm, they remained limited for larger column dimensions. Based on the analysis results, the combined influences of the cross-sectional dimension and longitudinal reinforcement ratio had no significant impact on the yield curvature (Figure 9).

For each considered axial load ratio, the equivalent yield curvature showed an increasing trend as the reinforcement ratio increased. Relative changes of up to 31% were determined, depending on the axial load level. It should be noted that despite the combined effect of the reinforcement ratio and cross-section diameter on the curvature response not being notable, the relative changes calculated for each *P*/*A_g_f_c_*′ level showed significant differences. In addition, the yield curvatures exhibited an increasing trend as the reinforcement ratio increased for the compressive strengths considered (Figure 9). 

The direct effect of the concrete compressive strength on the curvature response was determined to be less than 10% up to *P*/*A_g_f_c_*′ < 0.3. In contrast to this behavior, the combined effect of the axial load and concrete strength on the equivalent curvature was important for the further axial load levels and thus the equivalent curvatures rapidly decreased (Figure 9). This response exhibited similar characteristics for each column cross-section. The results in relation to the reinforcement ratio and concrete compressive strength are in excellent agreement with the results of various moment–curvature analyses conducted in the literature [14,15,17,19]. While the yield curvature had an increasing trend with the amount of longitudinal reinforcement ratio, the yield curvature decreased with the concrete strength, regardless of the yielding condition.

As a consequence of the moment–curvature analyses, it was deduced that the cross-section height and axial load level were the primary parameters influencing the equivalent yield curvature, as also indicated in references [14,15,21,22]. However, while the independent effects of the longitudinal reinforcement ratio and concrete compressive strength on the equivalent curvature were relatively limited, the combined effects of them with the axial load became important for the equivalent yield curvature. 

Even though the proposed simple equations may give reasonable results for low axial load levels, it is recommended that the curvature response be evaluated by moment–curvature analysis since the combined effects activated other responses at higher axial load levels, where the compressive concrete governed the yielding condition.

## Figures and Tables

**Table 1:** Summary of column models considered in the analyses.

Dimension *bxh* (cm)	Reinforcement Ratio *ρ_l_* (%)	Axial Load Level (*P/A_g_f_c_′*)	Concrete Comp. Strength *f_c_′* (MPa)	Yield Strength of Reinforcement *f_y_* (MPa)	Cover Thickness Ratio (*A_c_/A_g_*)
25 × 25 50 × 50 75 × 75 100 × 100 125 × 125 150 × 150 200 × 200	0.5 1.0 1.5 2.0 2.5 3.0 3.5 4.0	0 0.1 0.2 0.3 0.4 0.5	20 30 40 50 60	410	0.8
